# Probiotics Ameliorate Colon Epithelial Injury Induced by Ambient Ultrafine Particles Exposure

**DOI:** 10.1002/advs.201900972

**Published:** 2019-07-22

**Authors:** Xiaobo Li, Hao Sun, Bin Li, Xinwei Zhang, Jian Cui, Jun Yun, Yiping Yang, Li'e Zhang, Qingtao Meng, Shenshen Wu, Junchao Duan, Hongbao Yang, Jiong Wu, Zhiwei Sun, Yunfeng Zou, Rui Chen

**Affiliations:** ^1^ School of Public Health Advanced Innovation Center for Human Brain Protection Capital Medical University Beijing 100069 P. R. China; ^2^ Beijing Key Laboratory of Environmental Toxicology Capital Medical University Beijing 100069 P. R. China; ^3^ Key Laboratory of Environmental Medicine Engineering Ministry of Education School of Public Health Southeast University Nanjing 210009 China; ^4^ Department of Toxicology School of Public Health Guangxi Medical University Nanning Guangxi 530021 P. R. China; ^5^ Guangxi Colleges and Universities Key Laboratory of Prevention and Control of Highly Prevalent Diseases Guangxi Medical University Nanning Guangxi 530021 P. R. China; ^6^ Center for New Drug Safety Evaluation and Research China Pharmaceutical University Nanjing 211198 P. R. China; ^7^ School of Life Sciences Jiangsu Normal University Xuzhou 221116 China; ^8^ Institute for Chemical Carcinogenesis Guangzhou Medical University Guangzhou 511436 P. R. China

**Keywords:** air pollution, colonic epithelium, gut microbiota, *Lactobacillus*, ultrafine particles

## Abstract

Diesel exhaust particles (DEPs) are common airborne ultrafine particles (UFPs); however, few studies have examined their effects on the gastrointestinal tract. To investigate the interaction of gut microbiota and DEPs‐induced colonic injury, adult C57BL/6 mice are kept in whole‐body inhalation chambers and exposed to filtered room air (FRA) or DEPs (300 µg m^−3^) 1 h per day for 28 consecutive days. DEPs exposure results in colon epithelial injury with inflammatory cell infiltration and mucus depletion. Abundance of *Lactobacillus* in murine feces is transiently increased following 7‐day DEPs exposure and then decreased until the end of 28‐day exposure. A reduction of the colonic mucus layer thickness is observed in mice receiving gut microbiota from DEPs‐exposed mice. Mechanistically, RNA‐sequencing suggests disruption of the nitrogen metabolism pathway in DEPs‐exposed NCM460 cells. Upregulation of carbonic anhydrase 9 (CA9) expression levels is observed in epithelia following DEPs exposure both in vivo and in vitro. Oral administration of probiotics protects the mice against DEPS‐induced colon epithelial injury. The results strongly suggest the involvement of gut microbiota in response to DEPs exposure and subsequently epithelial injury in vivo. Supplementation with probiotic may be a potential way to protect against UFPs‐induced colon epithelial injury.

## 1 Introduction

Particulate matter (PM) pollution has been associated with pulmonary and extra‐pulmonary injuries for decades. Recent studies have reported effects on the gastrointestinal tract^1^, ^2^ and with an increased risk for appendicitis, digestive tract cancers, and inflammatory bowel disease (IBD).^3^ Intestinal exposure to PM occurs via mucocilliary clearance from the lungs and ingestion of contaminated food and water.^4^ The physiochemical characteristics and biological effects of airborne ultrafine particles (UFPs) are distinguishable from the lager airborne particles (such as fine particulate matter (PM_2.5_) and PM_10_).^5^, ^6^ As a major component of traffic pollution, diesel exhaust particles (DEPs) are airborne ultrafine particles (UFPs), with an aerodynamic diameter less than 100 nm.^7^ DEPs are comprised of organic and inorganic substances and have been considered a major contributor to the adverse health effects posed by ambient air pollutants.^8^

The gut microbiota is critical for human health^9^, ^10^ and maintains intestinal epithelial homeostasis offering a level of protection against gut injury.^11^, ^12^ Environmental stress can alter the gut microbiota and subsequently influence the host stress response.^13^ Animal models have demonstrated adverse intestinal effects following oral administration or pulmonary inhalation of PM. For example, in a low‐density lipoprotein receptor‐null (Ldlr^−/−^) mouse, oral ingestion of PM promoted lipid metabolism and inflammatory responses in small intestine and reduced the diversity of gut microbiota^2^, ^14^ and altered its composition.^15^, ^16^ Dysbiosis of the gut microbiota has been reported following PM inhalation^15^, ^16^ and associated with elevations in pro‐inflammatory cytokines in the murine colon.^17^ Specific for DEPs, intragastric administration to adult rats resulted in DNA damage and an oxidative stress response in colon epithelial cells.^18^ Ingestion of DEPs by mice promotes lipid metabolism, villus shortening, and inflammatory response in the small intestine.^2^ Above studies only reported the intestinal effects of DEPs via ingestion. So far, the effects of inhalational DEPs on colorectum, as well as gut microbiota, remain unknown.

In the current study, we examined the ability of inhaled DEPs to damage colonic epithelia and alter gut microbiota composition, as well as the potential protection of probiotics against DEPs‐induced colonic injury.

## 2 Results

### 2.1 Experimental Design

In the present study, we hypothesized that dysbiosis of gut microbiota was involved in the DEPs inhalation‐induced colon epithelial injury. We firstly observed the phenotype of epithelial injury and the alteration of gut microbiota composition in mice following DEPs inhalation; further, fecal microbiota transplant (FMT) suggested association between gut microbiota and epithelial injury. Secondly, an in vitro co‐culture model of bacteria and colon epithelial cells was used for RNA‐seq analysis, exploring the effects of gut bacteria on epithelia. Next, the expression levels of key molecules associated with DEPs exposure, as well as gut bacteria, were identified both in vitro and in vivo. Probiotics supplement to mice was used to observe the potential rescue against DEPs‐induced colon mucus depletion.

### 2.2 Inhalation of DEPs Led to Colon Epithelial Injury

**Figure 1:**
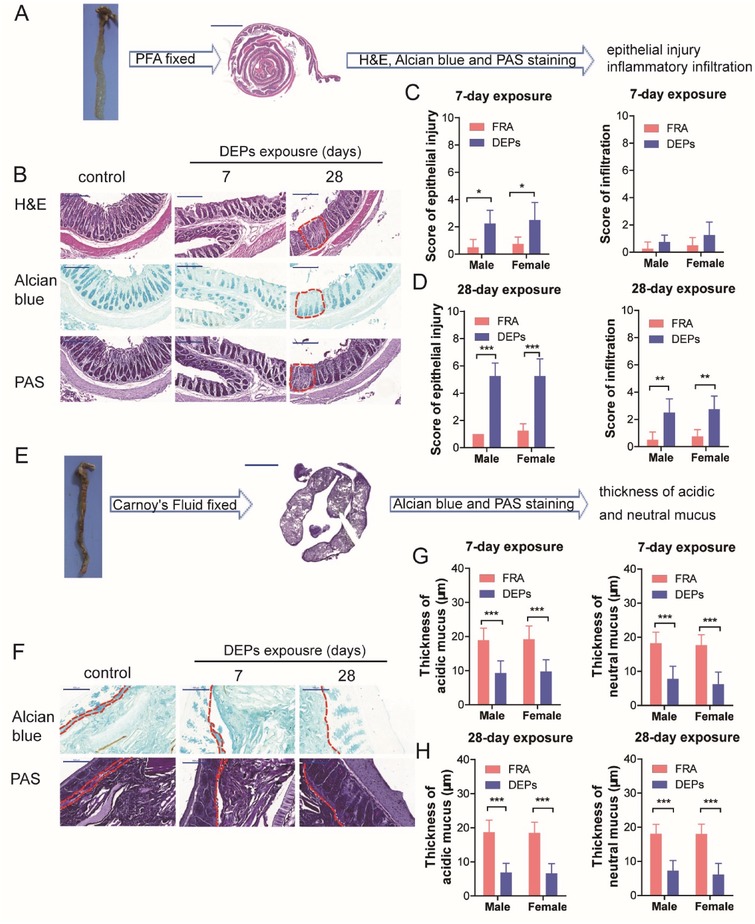
Inhalation of DEPs results in colon epithelial injury. A) Schematic diagram for the histopathological analysis using PFA fixed tissues (scale bar: 2000 µm). B) Representative pictures of H&E staining, Alcian blue staining, and PAS staining of the colonic tissue sections from mice exposed to FRA or DEPs for 7 or 28 days (scale bar: 200 µm). C) Epithelial injury scores and infiltration scores of murine colonic tissues following 7‐day DEPs exposure (*n* = 4 per sex per group, two‐way ANOVA). D) Epithelial injury scores and infiltration scores of murine colonic tissues following 28‐day DEPs exposure (*n* = 4 per sex per group, two‐way ANOVA). E) Schematic diagram for the pathohistological analysis using Carnoy's fluid fixed tissues (scale bar: 5000 µm). F) Representative pictures of Alcian blue staining and PAS staining of the colonic tissue sections from mice exposed to FRA or DEPs for 7 or 28 days (scale bar: 100 µm). G) Thickness of murine colonic mucus layer following 7‐day DEPs exposure (*n* = 18 [6 measurements/mouse × 3 mice per sex per group], two‐way ANOVA). H) Thickness of murine colonic mucus layer following 28‐day DEPs exposure (*n* = 18 [6 measurements/mouse × 3 mice per sex per group], two‐way ANOVA). *p < 0.05, **p < 0.01, and ***p < 0.001.

The mice were exposed to filtered room air (FRA) or DEPs for 28 consecutive days. Body weight was not found to be significantly different between DEPs‐ or FRA‐exposed groups at each time point. Next, we examined the histopathological alterations in murine colonic tissues including H&E, Alcian blue, and periodic acid–Schiff (PAS) staining on the Swiss‐roll sections (**Figure** **1**A). At day 7 of the exposure, colonic tissues from DEPs‐treated mice showed significant signs of epithelial injury but without dramatic infiltration of inflammatory cells as compared with FRA‐treated control mice (Figure 1B,C). Similar epithelial injury was observed in murine colonic tissues following 14‐ or 21‐day DEPs exposure (Figure S1, Supporting Information). The epithelial injury could be observed at proximal, middle, or distal colon sites, and no differences have been observed according to these three sites (data not shown). Until day 28 of the exposure, inflammatory infiltration was observed in DEPs‐exposed group and the epithelial injury scores went much higher as compared with FRA‐treated control mice (Figure 1B,D). Neither the interaction effects between DEPs exposure and sex nor the sexual difference following DEPs exposure were identified (Figure 1C,D). Additionally, we fixed the whole colonic tissues containing feces in Carnoy's fluid and performed Alcian blue and PAS staining (Figure 1E) to examine the thickness of mucus layer. Both acidic and neutral mucus was damaged by the inhalation of DEPs, which was seen as early as the 7th day of exposure (Figure 1F,G). The loss of acidic and neutral mucus lasted throughout the experimental period by showing reduction of the thickness of mucus layer (Figure 1F,H). Consistent with the pathological alterations, neither the interaction effects between DEPs exposure and sex nor the sexual difference following DEPs exposure was identified (Figure 1G,H). Thus, inhalation of DEPs damages colonic epithelium with loss of mucus and infiltration of inflammatory cells, in which loss of mucus occurred prior to inflammatory infiltration.

### 2.3 Inhalation of DEPs Led to Gut Microbiota Dysbiosis

**Figure 2:**
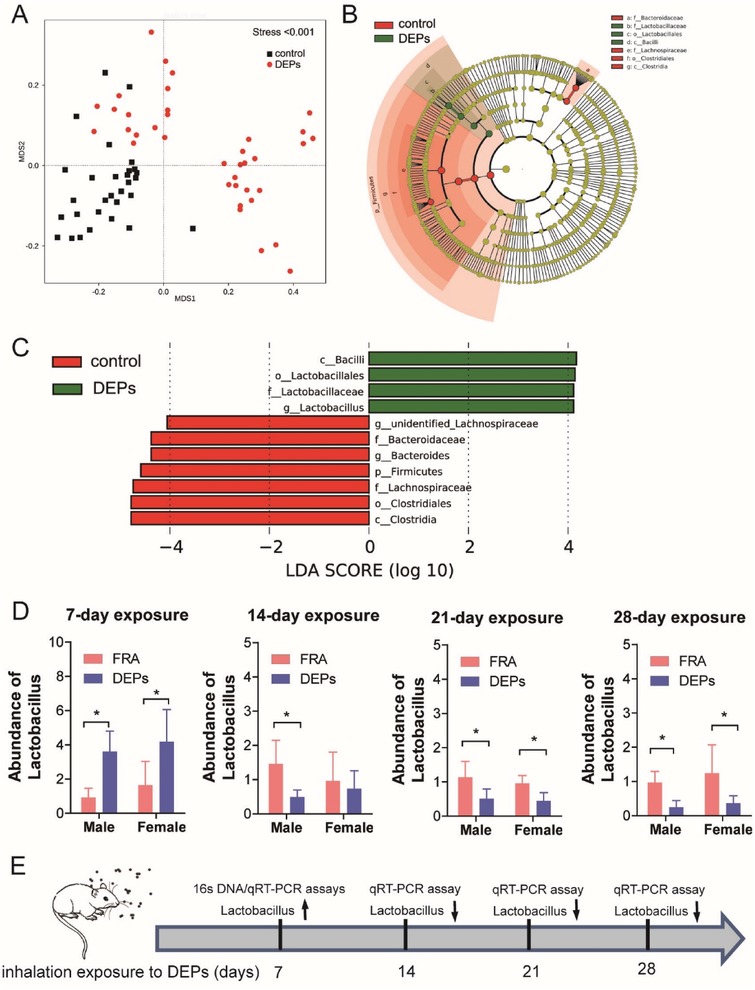
Inhalation of DEPs altered the composition of gut microbiota. A) Non‐metric multidimensional scaling analysis, B) LEfSe Cladogram, and C) LEfSe histogram of fecal microbiota profiling from DEPs group (fecal samples from male *n* = 20 and female *n* = 17) and FRA group (fecal samples from male *n* = 15 and female *n* = 15) following 7‐day exposure. D) qRT‐PCR analysis of the abundance of *Lactobacillus* in the stools from mice treated with DEPs or FRA. Stools were taken at the 7th, 14th, 21st, and 28th days of exposure (fecal samples *n* = 5 per sex per group, two‐way ANOVA, *p < 0.05). E) Summary of the alteration of *Lactobacillus* abundance in murine stools following DEPs exposure.

Intestinal mucus is secreted by intestinal goblet cells and the outer layer of colorectum mucus contains intestinal bacteria.^19^ Due to the reduction of mucus layer in the murine colons following 7‐day DEPs exposure, it is reasonable to explore whether the dysbiosis of gut microbiota occurs synchronously. We thus performed 16s rDNA sequencing to examine the alteration of fecal microbiota using stools collected on the 7th day of exposure. Non‐metric multidimensional scaling analysis showed that inhalation of DEPs clearly altered the composition of gut microbiota with the clusters of DEPs group distributed differently from that of FRA group (**Figure** **2**A). In depth analysis of data revealed that *Lactobacillus* was increased in the gut microbiota of DEPs‐treated mice (Figure 2B,C). Next, we measured the abundance of *Lactobacillus* in the stool samples, which were taken weekly post exposure, from the mice exposed to either DEPs or FRA by qRT‐PCR (Figure 2D). We found that *Lactobacillus* was transiently upregulated by the inhalation of DEPs for the 1st week but downregulated from the 2nd week post exposure and the decrease of *Lactobacillus* lasted throughout the rest of the experiment. There is neither interaction effects between sex and DEPs exposure nor the sexual difference identified of *Lactobacillus* abundance (Figure 2D,E). The results mentioned above suggested the dysbiosis of gut microbiota induced by DEPs inhalation. We hypothesized that *Lactobacillus* play a protective role in response to DEPs exposure, and the transient upregulation of gut *Lactobacillus* is a stress reaction to DEPs inhalation. However, the association between gut microbiota dysbiosis and colon epithelial injury remains unclear.

### 2.4 The Epithelial Injury was Driven by the Altered Gut Microbiota

**Figure 3:**
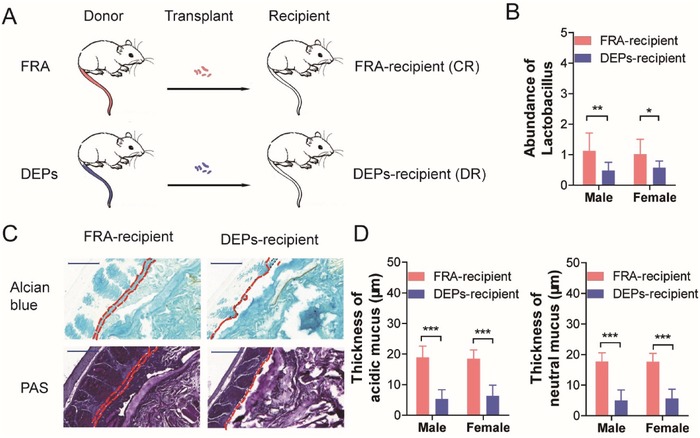
Effects of microbiota transplant on colon epithelial injury. A) Schematic of fecal microbiota transplant study design. B) qRT‐PCR analysis of the abundance of *Lactobacillus* in the stools from recipient mice (fecal samples *n* = 10 per sex per group, two‐way ANOVA). C) Representative images of Alcian blue and PAS staining of colonic tissues from FRA‐ or DEPs‐recipient mice (following 28‐day microbiota transplant). (scale bar: 100 µm.) D) Quantification of the thickness of mucus layer in the colonic tissues from recipient mice (following 28‐day microbiota transplant; *n* = 18 [6 measurements/mouse × 3 mice per sex per group], two‐way ANOVA). *p < 0.05; ^**^ *p* < 0.01; ^***^ *p* < 0.001.

To further validate the association between gut microbiota dysbiosis and mucus layer deterioration, we designed a fecal microbiota transplant experiment (FMT) using the feces from the mice treated with either FRA or DEPs. Schematic of the FMT experiment was shown in **Figure** **3**A. We measured the abundance of *Lactobacillus* in the feces from both FRA‐ and DEPs‐recipient mice on the 28th day of transplantation. As expected, in the feces from the DEPs‐recipient mice, the abundance of *Lactobacillus* was significantly lower than that of FRA‐recipient mice and no sex difference was identified (Figure 3B). We further performed Alcian blue and PAS staining to examine the thickness of mucus layer. Both acidic and neutral mucus were damaged in the DEPs‐recipient mice as compared to FRA‐recipient mice (Figure 3C,D). Therefore, DEPs‐induced dysbiosis of the gut microbiota triggered epithelial mucus depletion and thus could be one of the reasons for DEPs‐induced colon epithelial injury.

### 2.5 Molecular Pathways were Modulated in DEPs‐Exposed Epithelia In Vitro

**Figure 4:**
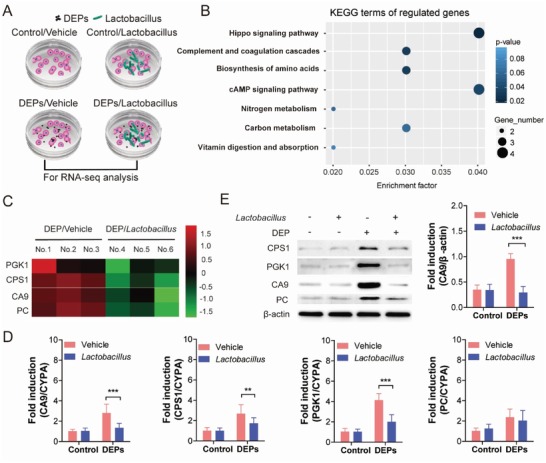
*Lactobacillus* prevented DEPs‐induced metabolic gene expression changes in colon epithelial cells. A) Schematic NCM460 and *Lactobacillus* co‐culture study design. B) KEGG enrichments of differently expressed genes. C) Heat map of selected differentially expressed metabolic genes (*PGK1*, *CPS1*, *CA9*, and *PC*) in the cells treated with DEPs/vehicle or DEPs/*Lactobacillus*. D) Validation of mRNA expression levels in the cells from four groups showed in Figure 4A by qRT‐PCR. (*n* = 6 per group, two‐way ANOVA.) E) Protein expression levels were validated in NCM460 cells by western blot. (*n* = 3 per group, two‐way ANOVA.) ^**^ *p* < 0.01, ^***^ *p* < 0.001.

Until now, the effects of *Lactobacillus* on the colonic epithelia following DEPs remain unknown; we therefore set a co‐culture model of *Lactobacillus* and colonic epithelia, with or without DEPs exposure. The lactic acid bacteria (LAB) existing in feces from C57BL/6 mice (DEPs‐exposed group on the 7th day of exposure) were cultured and isolated on de Man Rogosa and Sharpe (MRS) medium (Figure S2, Supporting Information). The 16s rDNA sequencing assay demonstrated that LAB isolated from murine feces belongs to *Lactobacillus*. Thus, this isolated *Lactobacillus* was used for co‐culture with NCM460 cells. The mRNA profiling of cells from two groups (DEP/vehicle‐ and DEP/*Lactobacillus*‐treated groups) were analyzed by RNA‐sequencing analysis (**Figure** **4**A), to explore the potential effects of *Lactobacillus* on DEPs‐treated epithelia. The genes of DEP/vehicle‐treated cells that had a fold change over 2.0 (compared with DEP/*Lactobacillus*‐treated group) and a FDR less than 0.05 were selected for further KEGG analysis. We found that *Lactobacillus* modulated multiple signaling pathways and metabolism‐related pathways in DEPs‐exposed NCM460 cells (pathway enrichment: *p* < 0.1) (**Table** **1** and Figure 4B).

**Table 1:** KEGG enrichment of significantly upregulated genes in DEPs/vehicle‐treated cells

Term^a^	*p*	Involved genes
hsa04390: Hippo signaling pathway	0.022	FZD10, CSNK1E, MYC, BMP6
hsa04610: Complement and coagulation cascades	0.031	CD55, CD46, F2R
hsa01230: Biosynthesis of amino acids	0.036	CPS1, PGK1, PC
hsa04024: cAMP signaling pathway	0.045	FOS, PDE3A, PDE4D, F2R
hsa00910: Nitrogen metabolism	0.067	CA9, CPS1
hsa01200: Carbon metabolism	0.076	CPS1, PGK1, PC
hsa04977: Vitamin digestion and absorption	0.086	SLC19A1, SLC19A2

^a^ ^)^Cut‐off for pathway enrichment: *p* < 0.1, compared with DEPs/*Lactobacillus*‐treated cells.

Gut microbiota interacts with colon epithelial cells and usually affects their metabolism.^20^ We also found enrichment of significantly modulated genes in metabolism‐related pathways, such as biosynthesis of amino acid, nitrogen metabolism, and carbon metabolism (Table 1 and Figure 4B). The metabolic signaling pathway‐involved genes included carbamoylphosphate synthase 1 (*CPS1*), phosphoglycerate kinase 1 (*PGK1*), carbonic anhydrase 9 (*CA9*), and pyruvate carboxylase (*PC*), which were significantly upregulated in DEPs/vehicle‐treated group, compared with DEP/*Lactobacillus*‐treated group (Figure 4C). We validated the mRNA expression levels of these 4 genes by qRT‐PCR and found that DEPs exposure significantly induced the mRNA expression of *PGK1, CA9*, and *CPS1* in NCM460 cells; however, co‐culture of *Lactobacillus* with NCM460 cells significantly inhibited the DEPs‐induced upregulation of these genes (Figure 4D). We further examined their protein expression levels by western blot. Co‐culture of *Lactobacillus* with NCM460 cells significantly inhibited the upregulation of CA9 and PGK1 induced by DEPs (Figure 4E and Figure S3, Supporting Information). Thus, *Lactobacillus* showed potential protection against DEPs‐induced disruption of metabolic pathway in vitro.

### 2.6 CA9 was Associated with DEPs‐Induced Colon Injury In Vivo

**Figure 5:**
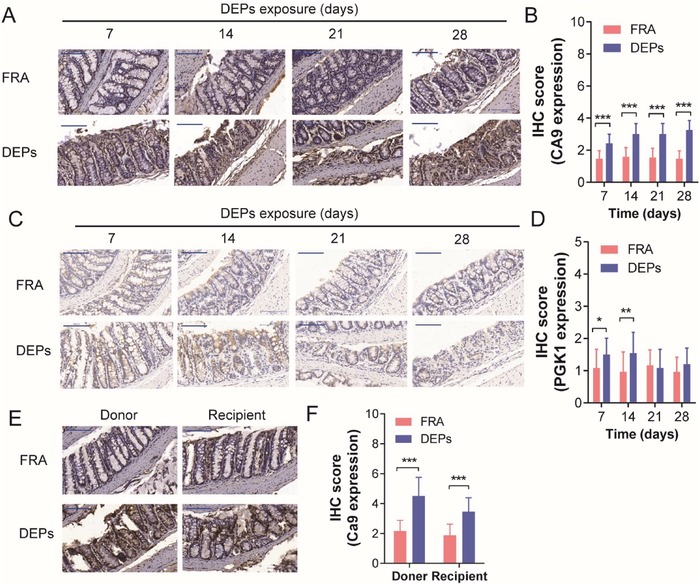
CA9 expression is enhanced in DEPs‐exposed murine colonic tissues. A) The protein expression levels of CA9 in colonic tissues (collected on the 7th, 14th, 21st, 28th day of exposure) were analyzed by IHC (scale bar: 100 µm). B) The IHC score of CA9 significantly increased in DEPs groups compared with FRA group across the whole experiment period (*n* = 24 [6 measurements per mouse × 4 mice per group], two‐way ANOVA). C) The protein expression levels of PGK1were analyzed by IHC. (Scale bar: 100 µm.) D) The IHC score of PGK1 significantly increased in DEPs groups compared with FRA group following 7‐ and 14‐day exposure (*n* = 24 [6 measurements per mouse × 4 mice per group], two‐way ANOVA). E) The protein expression levels of CA9 were analyzed in colornic tissues collected from the mice in FMT experiments by IHC. (Scale bar: 100 µm.) F) IHC score of CA9 were significantly increased in both DEPs‐donor and DEPs‐recipient groups, compared with FRA‐donor and FRA‐recipient groups, respectively (*n* = 24 [6 measurements per mouse × 4 mice per group], two‐way ANOVA). ^**^ *p* < 0.01, ^***^ *p* < 0.001.

To further explore the metabolism‐related gene expression in murine colons following DEPs inhalation, we performed immunohistochemistry (IHC) to examine the protein expression levels of CA9 and PGK1 in murine colonic epithelium in vivo. It was found that CA9 was upregulated in the colonic epithelium of DEPs‐treated mice throughout the experimental period (**Figure** **5**A,B), but PGK1 was only transiently upregulated for the first 2 weeks of the experiment (Figure 5C,D). Moreover, we examined the protein expression of CA9 in the colonic epithelium from the mice in the FMT experiment (described in Figure 3A). We found that the protein levels of CA9 in the colonic epithelium from the DEPs‐recipient mice were much higher than that from the FRA‐recipient mice, which mimicked the effects of DEPs inhalation (Figure 5E,F), indicating that the alteration of the expression of CA9 was caused by the dysbiosis of gut microbiota in DEPs‐treated mice. The results mentioned above suggested CA9 involved nitrogen metabolism pathway might be the common target for DEPs injury and *Lactobacillus* protection.

### 2.7 Probiotics Protects Mice from DEPs‐Induced Colonic Mucus Depletion

Our above results suggested the association between decreased abundance of *Lactobacillus* and colon epithelial injury, we next tried to explore if supplementation with probiotics could ameliorate DEPs‐induced colonic injury. We designed two studies, where the *Lactobacillus* isolated from murine feces or commercial probiotics (VSL#3) were orally administered to mice.

**Figure 6:**
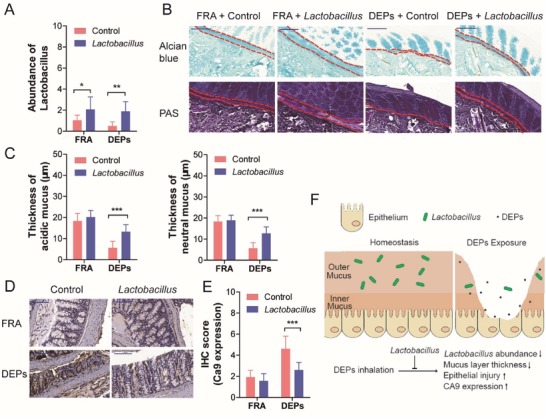
*Lactobacillus* administration protects the mice from DEPs‐induced colonic mucus depletion. A) qRT‐PCR analysis of the abundance of *Lactobacillus* in murine stool samples (fecal samples *n* = 10 per sex per group, two‐way ANOVA). B) Representative images of Alcian blue and PAS staining of colonic mucus layer. (Scale bar: 100 µm.) C) The thickness of mucus layer in murine colonic tissues (*n* = 18 [6 measurements per mouse × 3 mice per group], two‐way ANOVA). D) The protein expression levels of CA9 were analyzed by IHC. (Scale bar: 100 µm.) E) Supplementation of *Lactobacillus* significantly suppressed IHC score of CA9 expression in DEPs‐exposed group, compared with corresponding control (*n* = 18 [6 measurements per mouse × 3 mice per group], two‐way ANOVA). F) Schematic of molecular methanism involved in DEPs‐induced colon epithelial injury. *p < 0.05, ^***^ *p* < 0.001.

The mice were exposed to FRA or DEPs with or without *Lactobacillus* for 28 days, and then the abundance of fecal *Lactobacillus* was determined by qRT‐PCR. Oral *Lactobacillus* administration increased abundance of *Lactobacillus* in both FRA and DEPs‐treated mice (**Figure** **6**A). Next, the mucuses, including acidic and neutral mucus, were damaged by the inhalation of DEPs in the mice received vehicle (PBS) (Figure 6B,C). *Lactobacillus* supplementation protected the mice from the loss of both acidic and neutral mucus (Figure 6B,C). Finally, we measured the expression of CA9 in the murine colonic tissues. We found that in the mice treated with FRA, *Lactobacillus* supplementation alone did not alter the expression of CA9 in the colonic epithelium (FRA/*Lactobacillus* group); however, in the DEPs/*Lactobacillus* group, CA9 expression levels were significantly decreased, compared with DEPs‐exposed group (Figure 6D,E).

Next, the effects of commercial probiotics VSL#3 against DEPs‐induced colonic injury were evaluated. We found consistent results with the *Lactobacillus* supplementation, in which VSL#3 supplementation protected the mice from the loss of both acidic and neutral mucus following DEPs inhalation (Figure S4, Supporting Information). Taken together, our results suggest that DEPs‐induced dysbiosis of gut microbiota contributes to the colon epithelial injury and supplementation with probiotics can restore the decreased *Lactobacillus*, therefore, protected the mice from DEPs‐induced colonic mucus depletion (Figure 6F).

## 3 Discussion

In the present study, our results strongly suggest that dysbiosis of gut microbiota and the alteration of metabolic gene expression in colon epithelial cells are associated with DEPs‐induced colon epithelial injury. Probiotics supplementation may be a potential protection against DEPs‐induced injury.

Composition of gut microbiota has been reported to be altered following PM inhalation; however, the alterations of bacterial communities were not totally consistent across the literature. For example, following inhalational exposure to concentrated ambient particles (CAPs) for 3 weeks, microbiota analysis of murine fecal samples showed an increased abundance of unnamed genus with *Rikenellaceae*, *Bacteroidales*, and *Lachnospiraceae*, while abundances of *Staphylococcus*, *Lactobacillus*, and *Turicibacter* were decreased.^15^ Chronic inhalational exposure to CAP significantly increased the relative abundance of 9 bacterial taxa and decreased the relative abundance of 15 bacterial taxa in murine fecal samples.^16^ Increases in *Lachnospiracea* were observed by both Mutlu et al.^15^ and Wang et al.^16^ In our study, mice were exposed to DEPs and only the elevated abundance of *Lactobacillus* was identified following 7‐day exposure. All the three studies used the C57BL/6 murine model and it seems that long‐term PM exposure posed more severe dysbiosis in the gut microbiota. The difference among studies might be attributed to the period of exposure and the varied PM from different locations.

Gut microbiota has been widely studied and recognized to be involved in regulating the homeostasis of intestinal epithelium and contributing to pathophysiological processes.^10^, ^11^, ^12^, ^21^ Based upon the FMT experiment reported in the current study, we hypothesized that dysbiosis of gut microbiota is at least one of the causes for DEPs‐induced colon epithelial injury. In line with our RNA‐sequencing assay results, other studies strongly support the possibility that the gut microbiota is involved in metabolism and homeostasis of intestinal epithelium.^22^ Here, we found that CA9 was consistently upregulated in the colon epithelium from DEPs‐treated mice across the exposure period. Carbonic anhydrases (CA) function as enzymes catalyzing the reversible hydration of carbon dioxide into bicarbonate and protons,^23^ among which CA9 is controlled via the hypoxia‐inducible factor (HIF).^24^ Therefore, the increased expression of CA9 observed in the present study suggests potential alteration of intestinal microenvironment, such as hypoxia which may have been induced by DEPs. Furthermore, several studies focusing on increased intestinal CA9 expression suggested its association with the development of colorectal cancers,^25^, ^26^ implicating the long‐term effects of consistent elevated CA9 expression.

We found that *Lactobacillus* abundance increased during the early stage of exposure to DEPs. However, more prolonged DEPs exposure reduced the abundance of *Lactobacillus* in the gut microbiota, which may have contributed to damage to the colonic epithelium. We hypothesize that an increased abundance of *Lactobacillus* may serve to protect the colonic epithelium during the early period of exposure to DEPs. *Lactobacillus* is one of the commonly used probiotics in commercial products.^27^ Probiotics are increasingly used to prevent or treat a variety of intestinal diseases, including inflammatory bowel disease (IBD), acute infectious diarrhea, and antibiotic‐associated diarrhea.^28^ Here, we found that *Lactobacillus* administration effectively suppressed CA9 expression, protected against mucus depletion in murine colonic tissues following DEPs exposure. Consistent with our results, oral administration of probiotic bacteria could restore epithelial barrier integrity, colon physiologic function, and reduce mucosal inflammatory cytokine secretion.^29^ Therefore, our results suggest that supplementation of the diet with probiotics may provide some protection against DEPs‐related colonic injury. This possibility may have public‐health implications since it is not practical or economically feasible to eliminate DEPs exposure in the environment completely.

One limitation should be noted that 16s rDNA analysis also suggested several decreased bacterial taxa in gut microbiota following DEPs exposure; however, the potential function of these bacteria require further investigations.

## 4 Conclusion

In summary, our results provide evidence for the involvement of the gut microbiota in colon epithelial injury induced by inhalation to DEPs. Dysbiosis of gut microbiota triggered mucus depletion and subsequent epithelial damage, as well as inflammatory infiltration. Thus, manipulation of gut microbiota, such as treatment with probiotics, may potentially be useful in preventing or protecting against colonic injury caused by exposure to DEPs.

## 5 Experimental Section

*Animal Husbandry*: A total of 140 male and 84 female 8‐week old C57BL/6 mice (20–22 g) were purchased from SLRC Laboratory Animal Co., Ltd., (China) and housed in polycarbonate cages (4–6 mice per cage by sex) with corn cob bedding in a specific pathogen‐free (SPF) animal facility under a 12 h light/dark cycle, at a room temperature of 22.5 °C. Mice received rodent chow (Jiangsu Xietong Pharmaceutical Bio‐engineering Co., Ltd., China) sterilized by cobalt (Co) 60 radiation and autoclaved water ad libitum. Mice were allowed a 3‐day acclimation to the facility. All animals were treated humanely, with regard for alleviation of suffering, and experimental procedure was approved by the Committee on Animal Use and Care of Southeast University, China.

*Inhalational DEPs Exposure*: A total of 112 C57BL/6 mice were randomly assigned to FRA (male *n* = 28 and female *n* = 28) or DEPs (male *n* = 28 and female *n* = 28) groups. The mice in DEPs group were exposed to DEPs with a mean concentration of 300 µg m^−3^.^30^ The mice in control group received FRA at the same flow rate as the DEPs group. The mice were exposed to FRA or DEPs for 1 h per day (from 9:00 a.m. to 10:00 a.m.) during the experimental period for 28 days. Briefly, the experiment was conducted as follows. Mice in their original cages were kept in stainless‐steel Hinners‐type whole‐body inhalation chambers that were outfitted with air quality monitoring equipment and a dust aerosol generator (Beijing HuiRongHe Technology Co., Ltd., China). The concentration of dry DEPs (the Standard Reference Material 2975, National Institute of Standards and Technology, NIST, USA) aerosol was adjusted by the rotation speed of the rotary brush outfitted on the dust aerosol generator. The mass concentrations of DEPs aerosol in the whole‐body chamber were measured by a real‐time light scattering dust monitor (CEL‐712 Microdust Pro, CASELLA CEL Inc., USA). At the termination of each daily exposure, mice were removed from the chambers and maintained in their home cage.

*Histopathological Analysis*: Mice (seven males and seven females from each group) were randomly selected, weighted, euthanized under ether anesthesia following 7, 14, 21, or 28 days of exposure. Necropsies were performed and colonic tissues harvested. Four colonic tissues/sex/group were immersion fixed in 4% paraformaldehyde, and processed for paraffin embedding. Sections were rehydrated and stained by hematoxylin and eosin (H&E), Alcian blue, or Periodic Acid‐Schiff stain (PAS) for epithelial injury and inflammatory infiltration analysis. Three colorectal tissues/sex/group were immersion fixed in Carnoy's fluid to observe the integrity of colonic mucus layer by Alcian blue and PAS staining. Sections were scanned using Panoramic SCAN (3DHISTECH, Hungary) to obtain a whole slide image.

Each section was scored across the entire section for epithelial injury and inflammatory infiltration. Epithelial injury scores were calculated by summarizing the scores for gland mucodepletion (0, none; 1, mild; 2, moderate; 3, severe) and scores of tissue damage (0, no mucosal damage; 1, discrete epithelial lesions; 2, surface mucosal erosion or focal ulceration; 3, extensive mucosal damage and extension into deeper structures of the bowel wall). Inflammatory infiltration score was calculated by summarizing infiltration score (0, no inflammatory cells; 1, infiltration around crypt bases; 2, infiltration of muscularis mucosa; 3, infiltration of submucosa) and the percent area of each section (0, no involvement; 1, ≤25%; 2, ≤50%; 3, ≤75%; 4, ≤100%).

Scoring was conducted on three sites per sample (the proximal, middle, and distal colon, totally three measurements per sample) generating a final total score for each mouse. For epithelial injury or inflammatory infiltration, Panoramic Viewer software (3DHISTECH, Hungary) was used to measure the thickness of mucus. Two separate sites for proximal, middle, and distal colon (totally six measurements per mouse). Histological scoring of tissues was performed by an experienced pathologist blinded to experimental conditions.

*Bacterial 16s rDNA Sequencing*: Fecal pellets were collected daily (1 h after the cessation of DEPs exposure), stored into sterile tubes (≈0.02 g per tube), immediately frozen, and stored at −80 °C. Fecal samples from the mice in FRA (*n* = 15 male; *n* = 15 female) or DEPs (*n* = 20 male; *n* = 17 female) groups on the 7th day of exposure were used for microbiota composition measurement. DNA was extracted by the repeated bead beating plus column purification (The FastDNA SPIN Kit for Feces, MPBIO, USA) and then subjected to Miseq sequencing (Illumina, USA). The V3 and V4 hypervariable regions of the 16sRNA gene were amplified and sequenced following the 2 × 300 pair ended protocol. The DNA concentrations were measured using the Qubit dsDNA HS Assay Kit (Thermo Fisher Scientific, USA). The library preparation, sequencing, and data analysis were performed at Novogene Co. Ltd. (Beijing, China). Non‐metric multidimensional scaling (NMDS) analysis was used to identify the dissimilarities between FRA‐ or DEPs‐exposed gut microbiota matrixes. Linear discriminant analysis (LDA) effect size (LEfSe) analysis was used to identify the significantly differentiated bacterial taxa across samples with a cutoff LDA score >4.

*Fecal Lactobacillus Isolation and Culture*: Feces from C57BL/6 mice (DEPs‐exposed group on the 7th day of exposure) were mixed, and 0.1 g feces were homogenized with 1 mL sterile 0.9% saline. The homogenate was diluted and incubated on de Man Rogosa and Sharpe (MRS) (ELITE‐MEDIA, China) agar plate at 37 °C for 48 h under anaerobic condition. The lactic acid bacteria (LAB) colonies were isolated by sequential culture and identified based on the 16S rRNA sequencing (GenScript Biotech Corp., China). Sequence data were aligned and compared to the GenBank database.

LAB was cultured overnight at 37 °C in MRS broth (ELITE‐MEDIA, China). The culture was diluted 1:100 in fresh medium and cultured overnight under anaerobic condition. The optical density at 600 mn of culture medium was measured and the number of colony‐formation units (CFU) was determined according to standard growth curves. Samples were cultured overnight, LAB washed in phosphate‐buffered saline (PBS, pH7.4), and used co‐culture with NCM460 cells or immediately orally administered to the mice.

*Fecal Microbiota Transplant (FMT)*: In order to establish a cause‐effect relationship between the alteration of gut microbiota and the colon epithelial injury caused by the inhalation of DEPs, we performed FMT experiment using the stools from the mice (donors) either treated with FRA or DEPs. For donors, 8‐week‐old mice (20–22 g, seven males; seven females per group) were randomly assigned to receive FRA or DEPs following previously described exposure parameters. Starting from 1st day of exposure, the fecal microbiota of donor mice was collected and transferred to the age‐ and sex‐matched recipient mice (C57BL/6, 20–22 g, seven males and seven females per group) as previous reported.^31^ Briefly, the stools from control or DEPs donors were pooled in sterile saline (100 mg mL^−1^), resuspended by mixture, and centrifugalized at 800 ×*g* for 3 min. The supernatant was collected and delivered to the recipient mice via oral gavage (100 µL each recipient) within 10 min. The recipient mice were subjected to the microbiota transplant twice a week for 4 weeks. Before the transplantation, the recipient mice received ciprofloxacin (0.2 g L^−1^) and metronidazole (1 g L^−1^) (Sigma‐Aldrich, USA) in drinking water for 7 consecutive days

*Co‐Culture of NCM460 Cells and Lactobacillus*: The intestinal mucus layer serve as a protective barrier to intestinal epithelial cells and with depletion increased contact between epithelial and DEPs can occur.^32^ To examine the effect of DEPs on colon epithelial cells with or without *Lactobacillus*, we set up an in vitro model to study the gene expression profiles of NCM460 cells resembling the conditions of *Lactobacillus* co‐culture with or without DEPs treatment.

Human colon epithelial cells (NCM460; American Type Culture Collection) were cultured in Dulbecco's modified Eagle's medium (DMEM) supplemented with 10% fetal bovine serum (FBS), 100 U mL^−1^ penicillin, and 100 g mL^−1^ streptomycin and incubated at 5% CO_2_; 37 °C. Four experimental groups were established. 1) NCM460 alone (control [DMEM without serum and antibiotics]/vehicle [DMEM without serum and antibiotics to avoid sterilization of *Lactobacillus*]), NCM460 + *Lactobacillus* (control/*Lactobacillus*), NCM460 + DEPs (DEP/vehicle), and NCM460 + *Lactobacillus* + DEPs (DEPs/*Lactobacillus*). Co‐culture of NCM460 cells with *Lactobacillus* was performed as previous described.^33^ Briefly, *Lactobacillus* were washed twice with PBS and re‐constituted in DMEM (without serum and antibiotics). NCM460 cells (1 × 10^6^) were seeded in six‐well tissue culture plates and allowed to adhere overnight under incubated conditions. Then, either DMEM or 1 × 10^8^ bacterial cells were added to each well. DEPs were diluted with DMEM (without serum and antibiotics) and added to a final concentration of 0.12 µg cm^−2^.^34^ Cells were incubated in 5% CO_2_ at 37 °C for 1 h, then the supernatant removed, cells washed with PBS, total RNA and protein of cells from each group were harvested.

*RNA‐Sequencing Assay*: Total RNA was isolated from NCM460 cells (DEPs/vehicle or DEPs/*Lactobacillus*), using NReasy Kit (Qiagen China (Shanghai) Co Ltd, China) from three biological replicates. RNA‐sequencing was performed using Illumina HiSeq2000RNA sequencing (Illumina, San Diego, USA) by Novogene Co. Ltd. (Beijing, China). Differentially expressed genes (DEGs) were screened with a fold‐change (FC) cutoff of >2.0 and a false discovery rate (FDR) <0.05. KEGG enrichment over differentially expressed genes was performed using a functional annotation tool, Database for Annotation, Visualization, and Integrated Discovery (DAVID 6.7) with significance set at *p* < 0.01.

*qRT‐PCR Assay*: Total RNA was isolated from NCM460 cells (control/vehicle, control/*Lactobacillus*, DEPs/vehicle, and DEPs/*Lactobacillus*) using Trizol reagent (ThermoFisher Scientific, USA). RNA quality was confirmed by absorbance at 260 and 280 nm. One microgram RNA was reversed transcribed to cDNA using ReverTra Ace qPCR RT Kit (Toyobo, Japan). Then, the cDNAs were subjected to RT‐PCR analysis. mRNA expression levels of four genes (*CA9*, *CPS1*, *PGK1*, and *PC*) were detected. Gene expression was normalized to CYPA mRNA content. The primers used are listed in Table S1, Supporting Information.

*Western Blot Assay*: NCM460 cells (control/vehicle, control/*Lactobacillus*, DEPs/vehicle, and DEPs/*Lactobacillus*) were rinsed with PBS and lysed using RIPA buffer supplemented with 1% protease inhibitor cocktail (Millipore, USA). Total protein was determined by BCA protein assay (Beyotime, Shanghai, China) and then was used for western blot assay to detect the protein expression levels of CA9, CPS1, PGK1, and PC. BCA protein assay kit was used to determine the protein concentration. Twenty micrograms of protein samples were electrophoresed on a 10% SDS‐PAGE gel and then transferred to a PVDF membrane. Membranes were incubated overnight at 4 °C with antibodies (Abcam, USA) against phosphoglycerate PGK‐1 (1:100, ab38007), CPS‐1 (1:100, ab45956), PC (1:500, ab115579), CA‐9 (1:500, ab184006), or β‐Actin (1:10 000, ab8226, Abcam, USA) followed by incubation (1 h, RT) with HRP‐conjugated secondary antibodies. Protein bands were developed using Pierce ECL Western Blotting Substrate (ThermoFisher Scientific, USA). The protein band density was analyzed by Image J (National Institutes of Health, USA), and fold induction relative to β‐actin was used to show the expression levels of target protein.

*IHC*: Rehydrated sections of murine colorectal tissue (*n* = 4 per sex per group) were washed with 0.1 m TBS (pH 7.4), pretreated with 3% hydrogen peroxide for 30 min to quench endogenous peroxidase, rinsed, and incubated in a blocking solution (containing 3% FBS in TBS) for 30 min at RT. Sections were incubated with primary antibodies (Abcam) against CA‐9 (1:200, ab184006) or PGK‐1 (1:100, ab38007) overnight at 4 °C. Rinsed sections were incubated in biotinylated secondary antibodies (ZSJQ‐BIO Co. Ltd., Beijing, China) for 60 min at RT followed by avidin biotinylated‐HRP complex (ABC) solution by 60 min at RT and detected with 3‐diaminobenzidine (DAB; Sigma‐Aldrich).

Sections were scanned using panoramic SCAN (3DHISTECH, Hungary) to obtain images for examination by two experienced histologists using a semiquantitative immunohistochemistry score as previously reported.^35^, ^36^ Category A documented the intensity of immunostaining as 0–3 (0, negative; 1, weak; 2, moderate; 3, strong); category B calculated the percentage of immunoreactive area as 1 (0–25%), 2 (26–50%), 3 (51–75%), and 4 (76–100%). IHC score resulted from the multiplication of category A and B of each section.

Samples were examined from FRA‐ or DEPs‐exposed mice (on the 7th, 14th, 21st, and 28th day of exposure), FMT (on the 28th day of exposure for donor and microbiota transplant for recipient), or following *Lactobacillus* supplement or VSL#3 supplement experiments (on the 28th day of FRA‐ or DEPs‐exposure).

*Lactobacillus or VSL#3 Supplementation*: To explore if supplementation with probiotics could ameliorate DEPs‐induced colonic injury, mice concurrently exposed for 28 days to FRA or DEPs, received either sterile PBS, 2 × 10^9^ CFU *Lactobacillus* (isolated from murine feces), distilled water, or 5 × 10^9^ bacteria in the commercial probiotic reagent VSL#3 (*Lactobacillus casei, Lactobacillus plantarum, Lactobacillus acidophilus, Lactobacillus delbrueckii* subsp. *bulgaricus, Bifodobacterium longum*, *Bifidobacterium breve, Bifidobacterium infantis*, and Streptococcus salivarius subsp. *thermophiles*; Sigma‐Tau Pharmaceuticals, USA) in a dosing volume of 100 µL.

*Quantification of Fecal Lactobacillus by qPCR*: To determine the dynamic abundance of *Lactobacillus* following exposure, feces of mice in the inhalational DEPs exposure experiment collected on exposure day 7, 14, 21, or 28 (fecal samples *n* = 5 per sex per group) were examined.

To determine the abundance of *Lactobacillus* following fecal microbiota transplant (FMT), the feces were collected on exposure day 28 (fecal samples *n* = 10 per sex per group).

To determine the abundance of *Lactobacillus* following *Lactobacillus* or VSL#3 supplementation, the feces were collected on exposure day 28 (fecal samples *n* = 10 per sex per group).

Total bacterial DNA from feces was extracted using FastDNA Spin Kit for Feces (MP Biomedicals, Canada) and quantified using a spectrophotometer (Nanodrop 2000, Thermo Fisher Scientific, USA). Amplification was conducted with SYBR Green Qualitative Polymerase Chain Reaction (qPCR) Master Mix (BioRad, Hercules, CA) as previous study^37^ to detect the abundance of *Lactobacillus* in fecal samples.

*Statistical Analysis*: Data were presented as means ± SD unless indicated otherwise. For the comparison of multiple groups, two‐way ANOVA was used as indicated in the figure legends. The 2^−ΔΔCt^ method was used to analyze the qRT‐PCR results. The significance was set at *p* < 0.05.

## Conflict of Interest

The authors declare no conflict of interest.

## Supporting information

Supplementary
