# Supplementary material for: Probiotics Ameliorate Colon Epithelial Injury Induced by Ambient Ultrafine Particles Exposure
Source: Adv Sci (Weinh). 2019 Jul 22;6(18):1900972. doi: 10.1002/advs.201900972 (PMC6755525; doi:10.1002/advs.201900972)
Supplement: Supplementary file 1 — Supplementary [file ADVS-6-1900972-s001.pdf]

Copyright WILEY-VCH Verlag GmbH & Co. KGaA, 69469 Weinheim, Germany, 2019.

ADVANCED SCIENCE
Open Access

Supporting Information

for *Adv. Sci.,* DOI: 10.1002/advs.201900972

Probiotics Ameliorate Colon Epithelial Injury Induced by Ambient Ultrafine Particles Exposure

*Xiaobo Li, Hao Sun, Bin Li, Xinwei Zhang, Jian Cui, Jun Yun, Yiping Yang, Li'e Zhang, Qingtao Meng, Shenshen Wu, Junchao Duan, Hongbao Yang, Jiong Wu, Zhiwei Sun, Yunfeng Zou, and Rui Chen**

Copyright WILEY-VCH Verlag GmbH & Co. KGaA, 69469 Weinheim, Germany, 2019.

Supporting Information

Probiotics Ameliorates Colon Epithelial Injury Induced by Ambient Ultrafine Particles Exposure

*Xiaobo Li, Hao Sun, Bin Li, Xinwei Zhang, Jian Cui, Jun Yun, Yiping Yang, Li'e Zhang, Qingtao Meng, Shenshen Wu, Junchao Duan, Hongbao Yang, Jiong Wu, Zhiwei Sun, Yunfeng Zou, Rui Chen***

Figure S1 In halation of DEPs results in colon epithelial injury in mice.

(A) Representative pictures of H&E staining, Alcian blue taining and PAS staining of the colonic tissue sections from mice exposed to FRA or DPEs for 14 or 21 days (scale bar: 200 μm). (B) Epithelial injury scores of murine colonic tissues (n=4/sex/group, two-way ANOVA). (C) Infiltration scores of murine colonic tissues (n=4/sex/group, two-way ANOVA). ** P<0.01, *** P<0.001

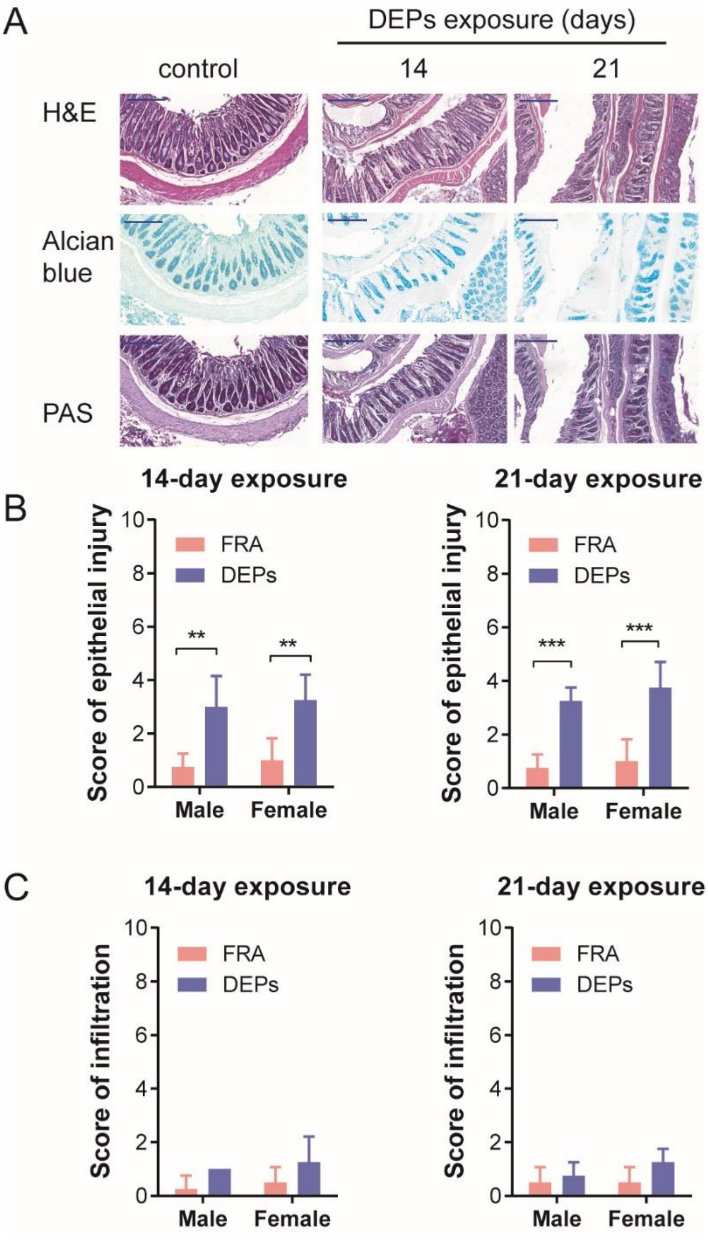

Figure S2 The lactic acid bacteria (LAB) in murine feces is isolated on de Man Rogosa and Sharpe (MRS) medium

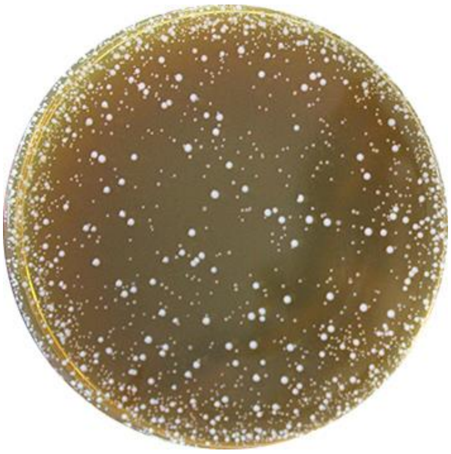

Figure S3 Protein expression levels in NCM460 cells.

Protein expression levels were validated in NCM460 cells by western blot. The fold induction for each protein was calculated according to intensity (n=3/group, two-way ANOVA). $^{*}P<0.05$, $^{**}P<0.01$

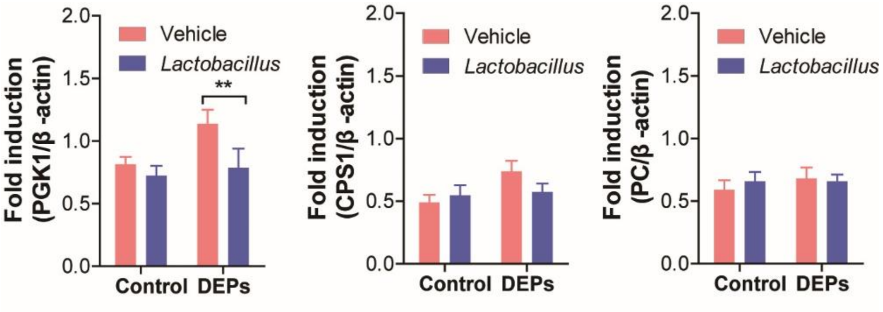

Figure S4 VSL#3 administration protects the mice from DEPs-induced mucus layer depletion. (A) Representative images of Alcian blue and PAS staining of colorectal mucus layer. (B) The thickness of mucus layer in murine colorectal tissues (n=18/group, two-way ANOVA). $^{***}P<0.001$

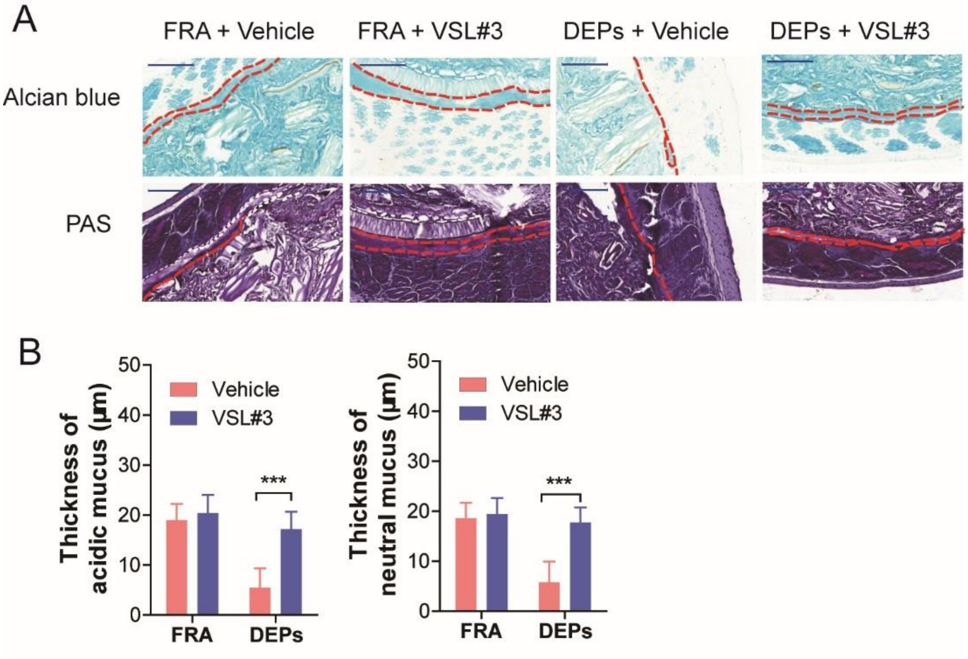

Table S1 The sequence of primers for human genes.

Gene symbol	Forward	Reverse
CA9	5'TTTGCCAGAGTTGACGAGGC3'	5'GCTCATAGGCACTGTTTTCTTCC3'
CPS1	5'AATGAGGTGGGCTTAAAGCAAG3'	5'AGTTCCACTCCACAGTTCAGA3'
PGK1	5' GAACAAGGTTAAAGCCGAGCC3'	5' GTGGCAGATTGACTCCTACCA3'
PC	5' GCTGGAGGAGAATTACACCCG3'	5' GGATGTTCCCATACTGGTCCC3'
